# Female-Inclusive Selective Versus Universal Neonatal Hip Ultrasonography Screening for Developmental Dysplasia of the Hip

**DOI:** 10.3390/children13091195

**Published:** 2026-09-04

**Authors:** Nina Đorđević, Sridhar Kalyanasundaram

**Affiliations:** 1 Orthopedic Department, Mediclinic Al Jowhara Hospital, Al Ain P.O. Box 14444, United Arab Emirates; 2 Pediatric Department, Mediclinic Al Jowhara Hospital, Al Ain P.O. Box 14444, United Arab Emirates; sridhar.kalyan@mediclinic.ae

**Keywords:** DDH, Graf method, female sex, neonates, ultrasonography, selective screening

## Abstract

**Highlights:**

**What are the main findings?**

- Female sex—although the strongest epidemiological determinant of DDH—has never been used as a primary screening criterion, and our data show the consequences of this omission.
- All dysplastic cases occurred in girls, including 40% without traditional risk factors, demonstrating that sex-based prevalence alone drives detection in many infants.

**What are the implications of the main findings?**

- Selective screening models that do not include all female neonates as a core eligibility group risk missing DDH cases, because female predominance is a consistent and well-established feature of DDH epidemiology.
- A female-inclusive selective screening pathway maintains DDH detection rates equivalent to universal screening while substantially reducing imaging volume.

**Abstract:**

Objectives: The objective of this study was to compare outcomes of universal versus female-inclusive selective ultrasonography screening for developmental dysplasia of the hip (DDH) in a neonatal population. Methods: This prospective cohort study evaluated two consecutive screening strategies at a single institution: a universal ultrasonography program (2019–2020; *n* = 354) and a selective program (2024–2025; *n* = 290). The selective protocol included all female neonates, breech presentation, positive family history, or abnormal clinical findings. All examinations were performed by the same operator using the Graf method. The primary outcome was detection of dysplastic hips; secondary outcomes included immature hip distribution, subgroup differences, and screening efficiency. Results: DDH detection rates were identical between cohorts (1.7%). In the universal cohort, six infants had dysplastic hips; in the selective cohort, five infants were affected, all females. Among these, 40% had no major risk factors. The proportions of mature and immature hips were comparable between cohorts. Screening efficiency was similar (number needed to screen: 59 vs. 58), despite a substantially reduced number of scans in the selective program. Conclusions: A selective screening program that includes all female neonates achieved DDH detection rates comparable to universal screening while significantly reducing imaging volume. As all dysplastic cases occurred in girls, this female-inclusive approach captured the highest-risk demographic without relying solely on traditional risk factors. Incorporating sex-based eligibility into selective screening may provide a safe and efficient framework for DDH screening programs in diverse clinical settings.

## 1. Introduction

Developmental dysplasia of the hip (DDH) is a major public health concern worldwide. Early detection remains critical, as timely intervention can prevent progression to severe deformity and reduce the burden of treatment.

Beyond its epidemiological burden, DDH carries serious long-term consequences if undiagnosed or inadequately treated. The most clinically relevant sequelae of late treatment include avascular necrosis, residual acetabular dysplasia, redislocation, and the subsequent need for reconstructive procedures, all of which determine long-term outcomes [1,2,3]. Although these complications are not the primary focus of the present study, they highlight the clinical importance of early diagnosis and reinforce the rationale for effective screening strategies.

Reported DDH incidence varies widely across populations and is strongly influenced by the screening strategy employed [4,5]. Universal ultrasonography programs aim to identify all cases early but may increase the detection of transient physiological immaturity. The Graf method, however, is calibrated to avoid overdiagnosis from the first days of life when performed by a trained and experienced examiner. Selective screening protocols, in contrast, target infants with predefined risk factors and can reduce imaging volume while maintaining diagnostic safety [6,7].

However, the effectiveness of traditional risk-based screening remains uncertain. The American Academy of Pediatrics (AAP) notes that commonly used risk factors—such as breech presentation or family history—are poor predictors of DDH, and that female sex alone accounts for approximately 75% of all cases [8]. Similarly, Roposch et al. demonstrated that established risk factors and even advanced prediction models have limited ability to reliably identify affected infants [9]. These findings highlight the limitations of relying solely on classical criteria and underscore the need to incorporate stronger predictors into selective screening strategies.

Female sex has consistently been reported as the risk factor for DDH [6,10,11], while other traditional risk factors such as breech presentation and family history show weaker predictive value [4,5]. Regional Gulf studies consistently demonstrate the same pattern, with a marked female predominance shaped by population-specific factors [12,13]. On the other hand, male infants have been shown to have less favorable outcomes following surgical management, underscoring the dual role of sex as both a risk factor and a prognostic determinant [14]. This makes sex-based considerations particularly relevant when designing screening protocols.

The present study was designed to evaluate the effectiveness of a selective screening strategy that includes all female neonates. We hypothesized that such a strategy would achieve detection rates comparable to universal screening while reducing the number of unnecessary ultrasonographic examinations. This hypothesis was tested in a prospective cohort using a standardized ultrasound protocol performed by a single experienced examiner.

## 2. Methodology

### 2.1. Study Design

This prospective cohort study was conducted at a single private hospital in the United Arab Emirates. Two consecutive neonatal hip ultrasonography screening protocols were evaluated: a universal screening program (2019–2020) and a selective screening program (2024–2025). Both cohorts were examined within the same institution using identical ultrasonography methodology and standardized Graf technique protocols performed by a single experienced operator.

### 2.2. Cohorts

The universal cohort included 354 consecutively screened neonates [10]. The selective cohort included 290 neonates who met predefined eligibility criteria. Based on strong evidence identifying female sex as the most significant independent risk factor for DDH, the selective protocol intentionally included all female neonates, regardless of additional risk factors. Other inclusion criteria were breech presentation, positive family history of DDH, or abnormal clinical findings. Neonates without any of these criteria were not referred for imaging under the selective protocol.

### 2.3. Screening Protocols

Ultrasonography was performed using the Graf method, with hips classified as mature (type Ia), immature (Type IIa), or dysplastic (Type IIc and above). In the universal cohort, all neonates were scanned in house at an average age of 3 days. In the selective cohort, newborns were referred by a neonatologist and scanned in the outpatient clinic at an average age of 14 days; 80% were still within the neonatal period. All neonates in both cohorts underwent clinical hip examination.

### 2.4. Data Collection and Outcomes

Prospectively collected data included sex, nationality, risk factors, and ultrasonography findings. The primary outcome was the detection rate of dysplastic hips. Secondary outcomes included the distribution of immature hips, subgroup differences by sex and nationality, and screening efficiency measured by the number needed to screen (NNS).

### 2.5. Statistical Analysis

Descriptive statistics were used to summarize demographic characteristics and ultrasonography findings. Detection rates and subgroup distributions were compared using counts and percentages. Screening efficiency was assessed by calculating the NNS. No inferential statistical testing was performed due to the small number of dysplastic cases.

### 2.6. Ethics

This project was conducted as a Quality Improvement (QI) initiative within Mediclinic Middle East and did not require formal ethics committee approval. All data were anonymized prior to analysis.

## 3. Results

### 3.1. Study Population

Demographic characteristics are summarized in Table 1. As expected, the proportion of females was higher in the selective cohort (82.8%) compared to the universal cohort (55.9%), reflecting the female-inclusive design of the program. Nationality subgroups included UAE nationals, other GCC nationals, and non-GCC residents.

### 3.2. Ultrasonography Findings

**Figure 1:**
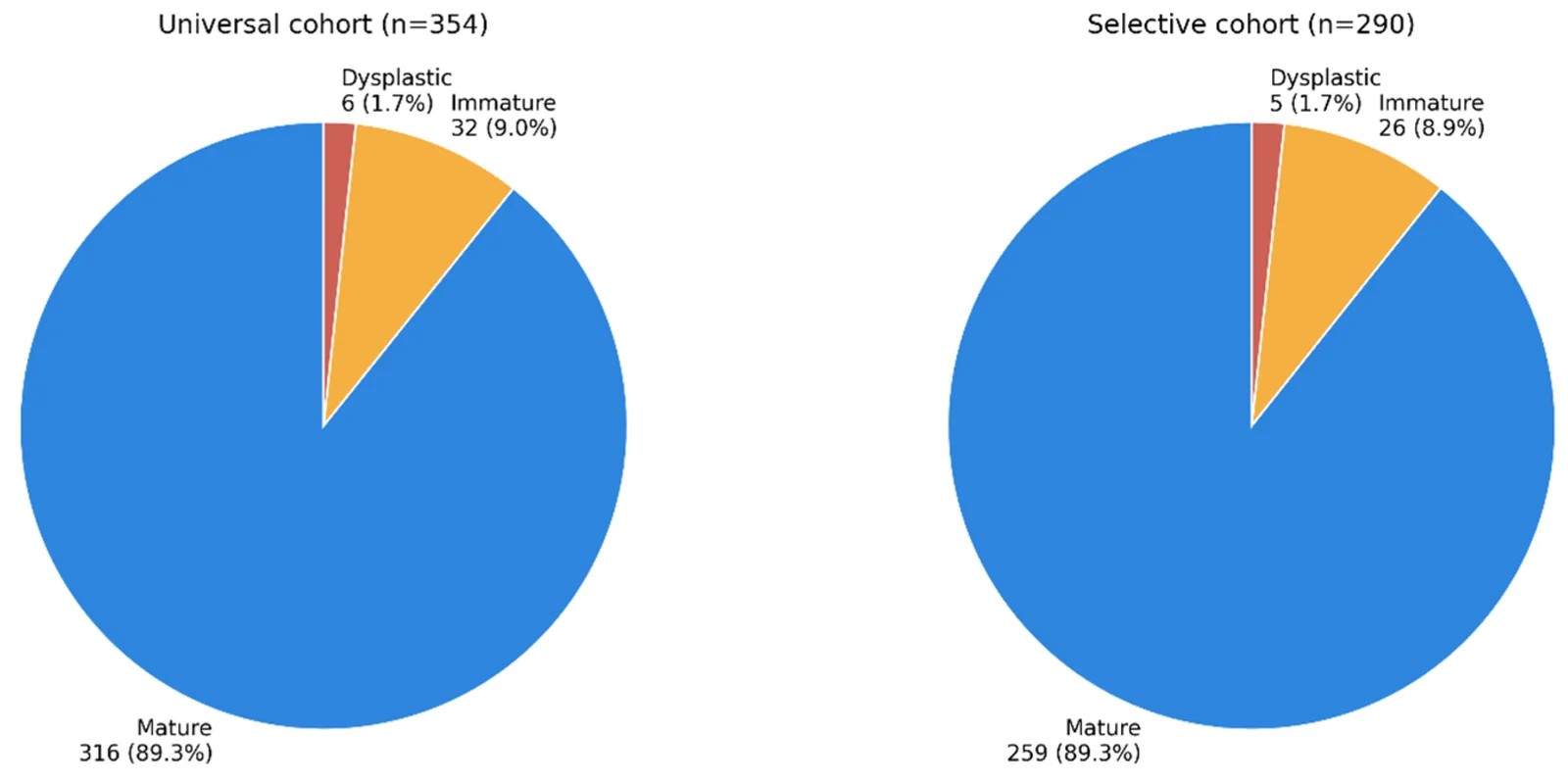
Ultrasonography outcome—cohorts’ comparison.

In the universal cohort, 316 neonates (89.3%) had mature hips, 32 (9.0%) had immature hips, and 6 (1.7%) had dysplastic hips [10]. In the selective cohort, 259 neonates (89.3%) had mature hips, 26 (8.9%) had immature hips, and 5 (1.7%) had dysplastic hips (Figure 1).

### 3.3. Nationality

Table 2 summarizes the incidence and distribution of DDH cases by sex and nationality.

### 3.4. Distribution of DDH Cases

In the previously published universal cohort (*n* = 354) [10], six infants (1.7%) were diagnosed with DDH. All but one were female. Among these, one had a breech presentation, while two others had a positive clinical finding—one demonstrated clinical laxity on left-positive Barlow/Ortolani (left hip was type III), and the other had a limited abduction of both hips (right was D and left was IIa). The two female DDH cases remaining were without any major risk factor (one was type IIC on the right and the other one was type D on the left). The single male case was identified as a type IIc dysplastic right hip in a 2 days old neonate from Morocco. He had no major risk factors.

In the selective cohort, all five infants diagnosed with DDH were UAE girls. Among these, one had a breech presentation (type III on left and limited abduction on examination were found at age of 45 days). The other two had a positive family history of DDH (a 33-day-old had a type IIc on the left with a limited abduction; and a 30-day-old had a type IIC on the right with a limited abduction), while none demonstrated clinical signs of hip instability at the time of referral. Two (40%) had no identifiable major risk factors (type IIC on the right at the ages of 12 and 19 days).

### 3.5. Efficiency Metrics

The number needed to screen (NNS) was 59 in the universal cohort and 58 in the selective cohort, indicating equivalent screening efficiency despite a substantially reduced number of scans in the selective program.

## 4. Discussion

This study compared two consecutive screening strategies for DDH implemented at the same institution: a universal ultrasonography program and a female-inclusive selective program. The four-year interval between cohorts was driven by financial constraints of the private health facility and provided a unique opportunity to evaluate a pragmatic adaptation of screening practice.

Because this study was cross-sectional, the conclusions are limited to the diagnostic yield of early neonatal screening. In Graf-based ultrasonography, dysplastic hips (type IIc and above) are identified at the time of screening, and only type IIa hips require follow-up to confirm maturation. Late-presenting DDH was therefore outside the scope of this analysis, although it remains the driving force behind efforts to implement more reliable screening programs and to avoid the numerous complications associated with delayed treatment [1,2,3].

The presence of DDH in infants lacking major risk factors is well recognized in the literature. Although breech presentation and family history are consistently reported as the strongest predictors of DDH, they do not account for all cases [5,8,9]. Large epidemiological studies and risk prediction models have shown that selective screening based solely on predefined criteria may miss affected infants, even within “at-risk” groups [4,9]. The findings of the present study align with this evidence and highlight the limitations of relying exclusively on risk-based eligibility in a population with diverse demographic characteristics.

Regional DDH research from the Gulf—predominantly from Saudi Arabia—demonstrates variability in incidence and risk factor patterns across regions and ethnic groups, influenced by factors such as consanguinity, traditional swaddling practices, and infant-care positioning customs [12,13]. These observations underscore the need for screening recommendations that reflect local epidemiology, rather than relying solely on criteria derived from Western populations where selective screening has historically been favored [6,8].

By including all girls—acknowledged as the dominant independent risk factor for DDH [8,9,10]—together with other established criteria, the selective program aimed to balance diagnostic yield with reduced imaging volume.

Consistent with established epidemiology, DDH occurred predominantly in females in both cohorts. All dysplastic cases in the selective cohort were identified in UAE girls. Within this subgroup, only one infant had a breech presentation and two had a positive family history, while none demonstrated clinical instability on neonatal examination. Notably, two of the five DDH cases (40%) had no identifiable major risk factors, meaning they would not have qualified for imaging under a traditional selective screening model [5,6]. Similar patterns have been described in other selective programs, where a substantial proportion of DDH cases occur in infants without recognized risk factors [3,6].

Our findings demonstrate that detection rates in the selective program were comparable to those achieved with universal screening, despite a lower number of scans performed. This supports the concept that female sex, when systematically incorporated, can serve as a strong filter in selective strategies, particularly in populations where female predominance is well documented. At the same time, the single male case detected in the universal cohort underscores the residual risk of sex-restricted screening and highlights the importance of cautious interpretation. At the same time, male sex has been reported as the least favourable prognostic factor for treatment outcomes, with poorer mid- to long-term results compared to females [14]. Taken together, these observations highlight that universal screening remains the only strategy capable of detecting all DDH cases. However, when financial or organizational constraints preclude universal implementation, it is preferable to base selective screening on the most significant epidemiological determinant—female sex—which captures the majority of cases, rather than relying solely on conventional risk factors that fail to identify a substantial proportion of affected infants. This compromise provides a pragmatic and resource-efficient pathway that maintains high detection rates, reduces unnecessary imaging, and aligns with the broader goal of minimizing late-presenting cases and their associated complications. Multicenter validation and long-term follow-up remain essential before widespread adoption of sex-restricted screening protocols can be recommended.

This study has several strengths, including the direct comparison of two screening pathways within the same institution and the inclusion of a large, diverse neonatal population. The use of a single operator and consistent ultrasonography methodology enhances internal validity.

This study has several limitations. The relatively small sample size and single-center setting limit generalizability. Because this study compares two non-contemporaneous cohorts, residual confounding related to temporal separation cannot be fully excluded. In addition, the lack of cost effectiveness analysis prevents evaluation of economic feasibility.

Although the number of dysplastic cases was small, limiting the ability to perform inferential statistical testing or multivariable analysis, the descriptive patterns observed—particularly the proportion of DDH cases without major risk factors—provide meaningful insight into screening performance and risk factor distribution. These findings are consistent with emerging international evidence [4,6,7,9]. In summary, detection rates were identical between the two pathways, but this equivalence must be interpreted in the context of the cohort’s sex distribution.

## 5. Conclusions

This study compared universal and female-inclusive selective neonatal hip ultrasonography screening pathways within a single institution and found comparable detection rates of DDH across both approaches. All dysplastic cases occurred in girls, consistent with the well-established female predominance in DDH epidemiology. Importantly, a substantial proportion of affected infants in the selective cohort had no major risk factors, indicating that their detection was influenced by sex-based prevalence rather than traditional selective criteria. These findings highlight that while sex strongly shapes DDH epidemiology, reliance on risk-factor-based screening alone may not reliably identify all affected infants. Screening strategies may therefore benefit from considering both the strong influence of sex and the limitations of conventional selective criteria when evaluating future approaches to early DDH detection. Confirmation in larger, multicenter studies with longer follow-up is required before changes to existing screening recommendations can be confidently implemented.

## Figures and Tables

**Table 1:** Demographic characteristics of the universal and selective screening cohorts.

Characteristic	Universal Cohort (*n* = 354)	Selective Cohort (*n* = 290)
Female, *n* (%)	198 (55.9)	266 (82.8)
Male, *n* (%)	156 (44.1)	24 (19.2)
UAE nationals, *n* (%)	142 (40.1)	169 (58.3)
Other GCC, *n* (%)	96 (27.1)	16 (5.5)
Non-GCC, *n* (%)	116 (32.8)	95 (32.7)

**Table 2:** Distribution of DDH by nationality and gender in the Universal and Selective cohorts.

Subgroup	Universal Cohort Dysplastic Hips	Selective Cohort Dysplastic Hips
Female	*n* = 5 (2.5%)	*n* = 5 (3.1%)
Male	*n* = 1 (0.6%)	*n* = 0 (0.0%)
UAE nationals	*n* = 3 (2.1%)	*n* = 5 (3.1%)
Other GCC	*n* = 2 (2.1%)	*n* = 0 (0.0%)
Non-GCC	*n* = 1 (0.9%)	*n* = 0 (0.0%)

## Data Availability

The data supporting the findings of this study are not publicly available due to patient confidentiality and institutional privacy regulations. De-identified data may be provided by the corresponding author upon reasonable request and subject to approval by Mediclinic Al Jowhara & Al Ain Hospitals.
